# Enforced OX40 Stimulation Empowers Booster Vaccines to Induce Effective CD4^+^ and CD8^+^ T Cell Responses against Mouse Cytomegalovirus Infection

**DOI:** 10.3389/fimmu.2017.00144

**Published:** 2017-02-20

**Authors:** Eleni Panagioti, Louis Boon, Ramon Arens, Sjoerd H. van der Burg

**Affiliations:** ^1^Department of Medical Oncology, Leiden University Medical Center, Leiden, Netherlands; ^2^Bioceros, Utrecht, Netherlands; ^3^Department of Immunohematology and Blood Transfusion, Leiden University Medical Center, Leiden, Netherlands

**Keywords:** cytomegalovirus, synthetic long peptides, prophylactic vaccination, T cells, OX40 costimulation

## Abstract

There is an imperative need for effective preventive vaccines against human cytomegalovirus as it poses a significant threat to the immunologically immature, causing congenital disease, and to the immune compromised including transplant recipients. In this study, we examined the efficacy of synthetic long peptides (SLPs) as a CD4^+^ and CD8^+^ T cell-eliciting preventive vaccine approach against mouse CMV (MCMV) infection. In addition, the use of agonistic OX40 antibodies to enhance vaccine efficacy was explored. Immunocompetent C57BL/6 mice were vaccinated in a prime-boost vaccination regiment with SLPs comprising various MHC class I- and II-restricted peptide epitopes of MCMV-encoded antigens. Enforced OX40 stimulation resulted in superior MCMV-specific CD4^+^ as CD8^+^ T cell responses when applied during booster SLP vaccination. Vaccination with a mixture of SLPs containing MHC class II epitopes and OX40 agonistic antibodies resulted in a moderate reduction of the viral titers after challenge with lytic MCMV infection. Markedly, the combination of SLP vaccines containing both MHC class I and II epitopes plus OX40 activation during booster vaccination resulted in polyfunctional (i.e., IFN-γ^+^, TNF^+^, IL-2^+^) CD4^+^ and CD8^+^ T cell responses that were even higher in magnitude when compared to those induced by the virus, and this resulted in the best containment of virus dissemination. Our results show that the induction of strong T cell responses can be a fundamental component in the design of vaccines against persistent viral infections.

## Introduction

It is estimated that 60–80% of people worldwide are infected by the prototypic β-herpesvirus human cytomegalovirus (HCMV). CMV establishes low-level viral persistence within immunocompetent hosts without clinical symptoms. However, it can cause life-threatening disease in the immunological immature (unborn babies and newborns) and immunocompromised individuals (e.g., bone marrow and organ transplant recipients) (1, 2). Although new antiviral drugs against CMV are in clinical development, the most commonly used agents display toxicity. Importantly, no licensed prophylactic or therapeutic vaccines exist for CMV at present. Consequently, there is an imperative need to identify potent vaccine modalities to prevent HCMV infection (3–5).

CD4^+^ and CD8^+^ T cell responses play a critical role in controlling CMV infection in both mouse and human. While CD4^+^ T cells seem to be more crucial in the early phase after infection, CD8^+^ T cells are imperative during latency and harbor superior protective properties upon rechallenge (6–9). Moreover, adoptive transfer approaches established the pivotal role of CMV-specific CD4^+^ and CD8^+^ T cells in orchestrating virus replication control (10–12). During CMV infection, CD4^+^ and CD8^+^ T cell responses either follow the traditional course comprised by massive expansion followed by rapid contraction and maintenance at low levels or instead do not undergo contraction but remain at high frequency or even expand gradually. The latter has been described as memory T cell inflation and has been observed for a restricted set of immunodominant CMV antigens (13–16). Memory inflation is thought to occur due to low-level persistent antigenic priming and requires certain costimulatory receptor-ligand pairs of which CD27–CD70 and OX40–OX40L interactions are important (17, 18). Phenotypically inflationary T cells exhibit effector-like properties without signs of exhaustion (14, 16, 19). The comparable nature of the T cell response to mouse CMV (MCMV) and HCMV is also found for B cell and NK cell responses and is likely related to the similarities of both viruses in tropism, pathology, and the establishment of latent infection that reactivates upon immunosuppression (20, 21).

Recently, we demonstrated that synthetic long peptide (SLP) vaccines, designed to exclusively induce MHC class I-restricted CD8^+^ T cells, were able to elicit robust and polyfunctional T cell responses that led to reduced MCMV replication in C57BL/6 and BALB/c mouse strains after challenge (22). However, live MCMV vaccines were more efficient which prompted us to further improve the SLP vaccine efficacy. Since CD4^+^ helper T cells promote long-term maintenance of memory CD8^+^ T cells, also during MCMV infection (23, 24), and display direct antiviral capabilities (12, 25), the induction of CD4^+^ T cells may improve the efficacy of the SLP vaccine. Here, we analyzed the potency of SLP vaccines inducing MCMV-specific CD4^+^ T cells, either alone or in conjunction with SLPs eliciting MCMV-specific CD8^+^ T cells. Enforced OX40 signaling was used to enhance the expansion of both CD4^+^ and CD8^+^ T cell subsets. We show that combined administration of SLPs eliciting CD4^+^ and CD8^+^ T cells and OX40 stimulation during booster vaccination leads to a startling increase of both the T cell magnitude and polyfunctionality, ultimately leading to efficient control of lytic MCMV infection.

## Animals and Methods

### Mice

Wild-type female C57BL/6 mice (8–10 weeks) were purchased from Charles River Laboratories (L’Arbresle, France) and Ly5.1 (SJL; CD45.1) congenic mice on a C57BL/6 genetic background were obtained from The Jackson Laboratory. Mice were bred and housed under specific-pathogen-free conditions at the Central Animal Facility of Leiden University Medical Center (LUMC). Experimental procedures were approved by the LUMC Animal Experiments Ethical Committee and conducted according to the Dutch Experiments on Animals Act and the Council of Europe (#13156 and #14187).

### Viral Infections

Virus stocks were prepared from salivary glands of BALB/c mice infected with MCMV-Smith [American Type Culture Collection (ATCC)]. The viral titers of the produced virus stocks were determined by viral plaque assays with mouse NIH-3T3 Embryonic Fibroblasts (ATCC). C57BL/6 mice were infected intraperitoneally (i.p.) with 5 × 10^4^ PFU MCMV in 400 µl of PBS. 60 days post-booster vaccination or infection, mice were rechallenged with 5 × 10^4^ PFU MCMV. Viral loads in spleen, liver, and lungs were determined by real-time PCR at day 5 post challenge as described previously (26). Due to differences in peak viral replication, the viral load in the salivary glands was not measured.

### Peptides and Vaccination

Short (9–10 aa) and long (20–21 aa) peptides containing MHC class I-restricted T cell epitopes (22) and 15 aa long peptides containing MHC class II epitopes (15) were produced at the GMP-peptide facility of the LUMC. The purity (75–90%) of the synthesized peptides was determined by HPLC and the molecular weight by mass spectrometry. All peptide sequences used in this study are listed and described in Table S1 in Supplementary Material. Both single and mixed SLP vaccines were administered subcutaneously (s.c.) at the tail base by delivery of 50 µg of each SLP and 20 µg CpG (ODN 1826, InvivoGen) in a total volume of 50 µl in PBS. Booster SLP vaccinations were provided after 2 weeks. At the indicated times (during prime and/or booster vaccination), mice were injected i.p. with 150 µg agonistic OX40 mAb (clone OX86) dissolved in 150 µl of PBS. All SLP vaccine administrations were well tolerated without adverse events and signs of hypersensitivity.

### Flow Cytometry

To evaluate CD4^+^ and CD8^+^ T cell responses, cell surface and intracellular cytokine staining in splenocytes and blood lymphocytes were performed as previously described (27). In brief, to determine the cytokine production capacity, single-cell suspensions from spleens were stimulated with short MHC class I peptides (2 µg/ml) for 5 h in the presence of brefeldin A (Golgiplug; BD Pharmingen) or with long MHC class II peptides (5 µg/ml) for 8 h of which the last 6 h in presence of brefeldin A. MHC class I tetramers specific for the M45_985–993_, M57_816–824_, m139_419–426_, M38_316–323_, and IE3_416–423_ MCMV epitopes were used. Fluorochrome-conjugated mAbs were obtained from BD Biosciences, Biolegend, or eBioscience. Flow cytometry gating strategies are shown in Figure S6 in Supplementary Material. All data were acquired on a LSRFortessa cytometer (BD Biosciences) and analyzed with FlowJo-V10 software (Tree Star).

### Antibody Detection by ELISA

Total IgG, IgG_2b_, IgG_2c_, IgG_3_, IgE, and IgA concentrations were determined by ELISA in serum samples as previously described (26). Briefly, Nunc-Immuno Maxisorp plates (Fisher Scientific) were coated overnight with 2 µg/ml MHC class II SLPs in bicarbonate buffer, and after blocking (skim milk powder, Fluka BioChemika) sera from mice (i) chronically infected, (ii) long term vaccinated with MHC class II SLP vaccines and anti-OX40 mAb treated (i.p. during booster vaccination only), or (iii) uninfected were added. Next, plates were incubated with various HRP-conjugated antibodies (SouthernBiotech) to detect different immunoglobulin isotypes. Plates were developed with TMB substrate (Sigma Aldrich), and the color reaction was stopped by the addition of 1 M H_2_SO_4_. To serve as a positive control, a peptide from the M2 protein (eM2) of influenza A virus with known ability to induce antibodies and corresponding serum was used. Optical density was read at 450 nm (OD_450_) using a Microplate reader (Model 680, Bio-Rad).

### Adoptive T Cell Transfers

The secondary expansion potential of the SLP vaccine-induced antigen-specific CD8^+^ T cells receiving agonistic OX40 mAb only during booster vaccination was determined by adoptive transfers. Splenic memory (day 65) CD8^+^ T cells from SLP vaccinated CD45.1^+^ congenic mice were negatively enriched with magnetic sorting using the CD8^+^ T cell isolation kit (Miltenyi Biotec). 2 × 10^6^ total CD8^+^ T cells were retro-orbitally injected (in a total volume of 200 µl in PBS) into naive CD45.2^+^ recipients. Recipient mice were rested for 2 h and concomitantly infected with 5 × 10^4^ PFU MCMV. Subsequently, in order to quantify the number of the vaccine antigen-specific CD8^+^ T cells that was transferred, a representative amount of cells was stained with MHC class I tetramers and with fluorochrome labeled antibodies against CD44, CD3, CD4, and CD8. The number of the donor’s vaccine-specific T cells transferred was ranging between 8 × 10^3^ and 2.5 × 10^4^ cells for the group that did not receive OX40 mAb and between 1.8 × 10^4^ and 4.5 × 10^4^ cells for the OX40 mAb boosted group. 6 days later, the number of vaccine-specific CD8^+^ T cells of the donor was measured (based on the expression of the CD45.1 marker) by flow cytometry, and the fold expansion was calculated.

### Statistical Analyses

Statistics were calculated using the unpaired Student’s *t*-test or ANOVA in GraphPad Prism software (GraphPad Software Inc., USA). *P < 00.5, **P < 0.01, ***P < 0.001, and ****P < 0.00001.

## Results

### Prime/Boost Vaccination with SLPs Inducing MCMV-Specific CD4^+^ T Cell Responses

**Figure 1:**
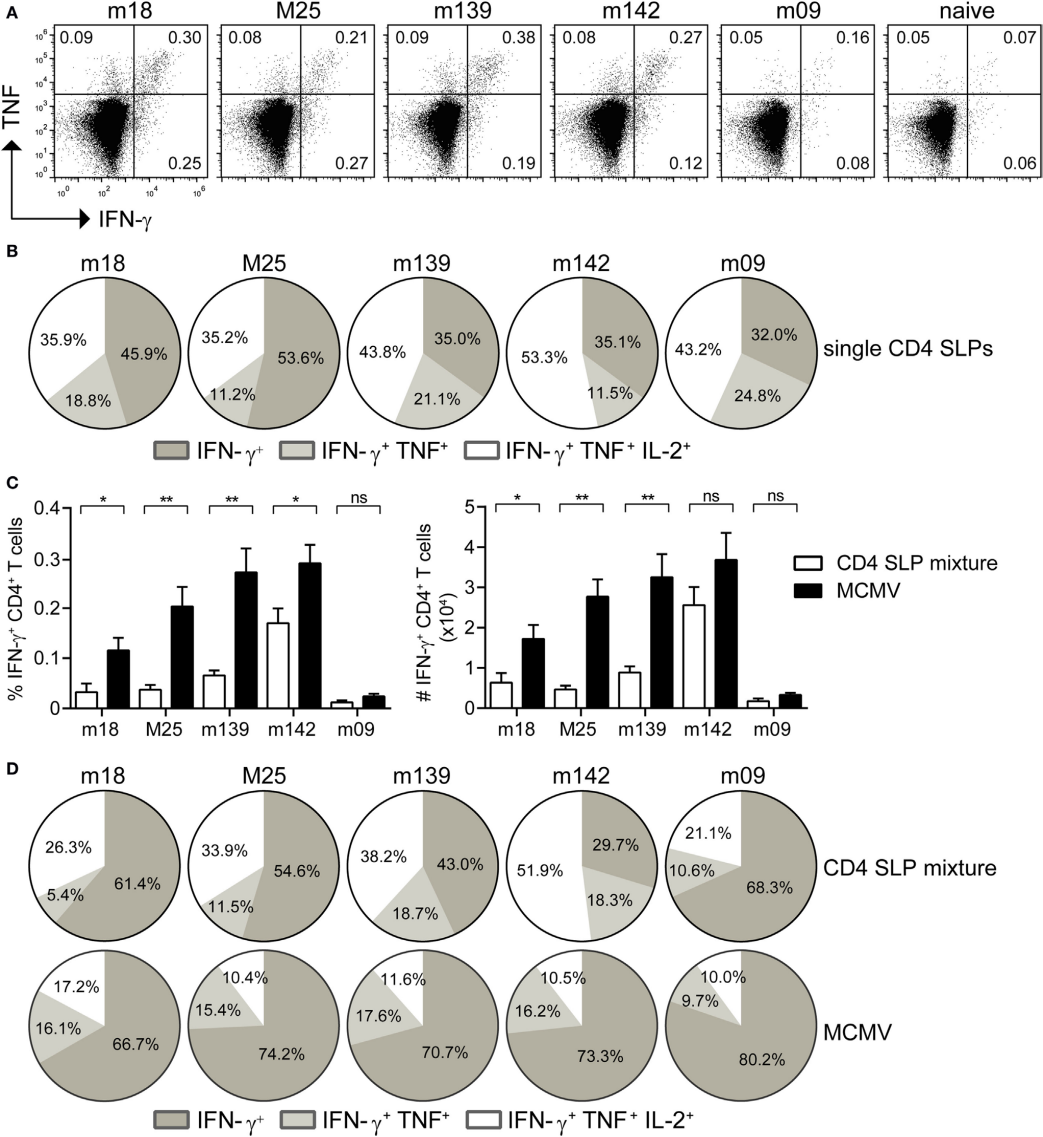
**Prime-boost synthetic long peptide (SLP) vaccination with combined MHC class II SLPs provokes the induction of strong and polyfunctional CD4^+^ T cell responses**. **(A)** At day 8 post-booster vaccination with single SLPs containing MHC class II epitopes, the magnitude of the CD4^+^ T cell responses specific to the indicated epitopes was determined by intracellular cytokine staining after restimulation with peptide. Representative plots show percentages of IFN-γ versus TNF cytokine production of the vaccine-elicited CD4^+^ T cells. **(B)** Pie charts depict the percentages of the single (IFN-γ), double (IFN-γ/TNF), and triple (IFN-γ/TNF/IL-2) cytokine producers of each antigen-specific CD4^+^ T cell population at day 8 post-booster vaccination with single MHC class II SLPs. **(C)** Percentages (left) and total numbers (right) of splenic SLP and MCMV-specific IFN-γ^+^ CD4^+^ T cells at day 8 post-booster vaccination with a mixture of all five MHC class II SLPs are shown. **(D)** Pie charts depict the percentages of the single (IFN-γ), double (IFN-γ/TNF), and triple (IFN-γ/TNF/IL-2) cytokine producers of each antigen-specific CD4^+^ T cell population at day 8 after booster vaccination with a mixture of all five MHC class II SLPs. Data represent mean values and are representative of three independent experiments (*n* = 4–5 per group). *P < 0.05; **P < 0.01; ns, not significant.

MHC class II epitopes from MCMV-encoded proteins have previously been identified by us in the C57BL/6 mouse strain (MHC haplotype H-2^b^) (15), and five immunogenic epitopes (i.e., m18_872–886_, M25_409–423_, m139_560–574_, m142_24–38_, and m09_133–147_) were selected for the SLP vaccine platform (Table S1 in Supplementary Material). Initially, the potential of single SLP-based vaccines in eliciting CD4^+^ T cell responses was assessed in a prime/boost vaccination setting (2 weeks apart) with the TLR9 ligand CpG as adjuvant. At day 8 after the first SLP vaccination, CD4^+^ T cell responses were not detected by polychromatic intracellular cytokine staining (data not shown) but they became detectable in the spleen at day 8 after the booster SLP vaccination (Figure 1A). However, these responses were relatively low when compared to SLP-induced CD8^+^ T cell responses (22). Analysis of the cytokine profile revealed the presence of single IFN-γ, double IFN-γ/TNF, and triple IFN-γ/TNF/IL-2 cytokine-producing CD4^+^ T cell populations (Figure 1B).

For clinical applications multiple SLPs need to be combined in order to deal with MHC heterogeneity. Moreover, the breadth of SLP vaccines is important for the efficacy (22). Hence, mice were vaccinated with a mixture of the five SLPs, and the CD4^+^ T cell response for each individual peptide epitope was measured. CD4^+^ T cell reactivity to all MHC class II epitopes was detected (Figure 1C), albeit that the response to each individual peptide was lower when compared to single SLP vaccination, suggesting that competition among CD4^+^ T cell peptide epitopes occurs in multivalent vaccines. Moreover, in comparison to the CD4^+^ T cell response observed after MCMV infection, vaccination with a mixture of MHC class II SLPs resulted in lower numbers of MCMV-specific T cells (Figure 1C). Nevertheless, the cytokine polyfunctionality of the SLP-elicited CD4^+^ T cells was augmented compared to MCMV-induced CD4^+^ T cells (Figure 1D). Taken together, these results show that prime/boost vaccination with a mixture of MHC class II epitope-containing SLPs elicits polyfunctional MCMV-specific CD4^+^ T cell responses, but in magnitude these are lower as compared to those induced by the virus itself.

### Enforced OX40 Triggering during Booster Vaccination Shows Superior Induction of SLP-Elicited CD4^+^ T Cell Responses

**Figure 2:**
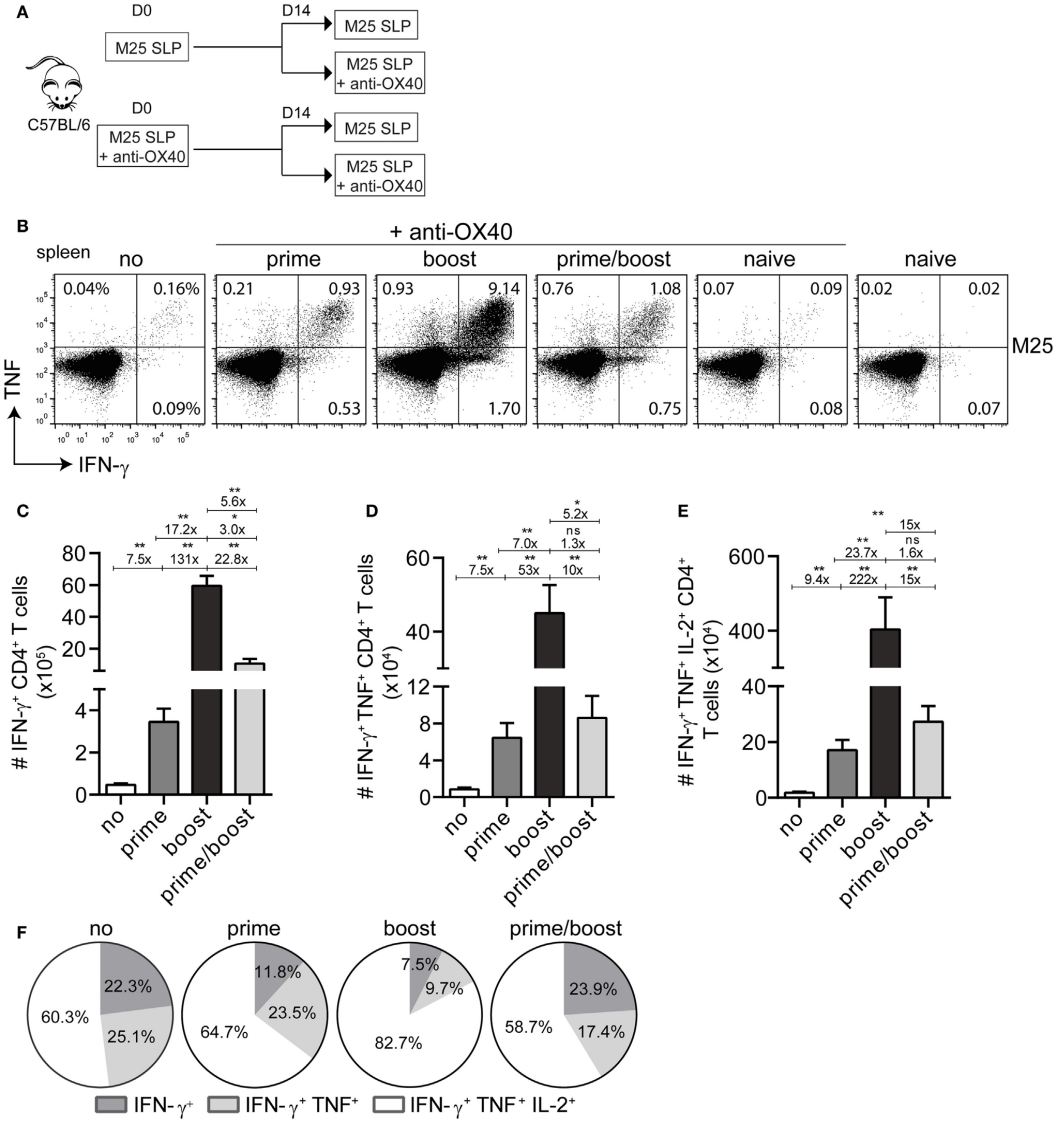
**Activation of the OX40 axis during booster vaccination with a single MHC class II synthetic long peptide (SLP) vaccine propels increment of the vaccine-induced CD4^+^ T cell response**. **(A)** Scheme of the experimental procedure and the vaccination timeline. Wild-type C57BL/6 mice were vaccinated (i) s.c. with M25_409–423_ MHC class II SLP alone or (ii) with M25_409–423_ MHC class II SLP (s.c.) along with anti-OX40 mAb (i.p.). Two weeks after prime vaccination mice from group (i) and (ii) were divided into two groups, respectively, and a booster immunization was administered. Half mice received only the M25_409–423_ SLP and the other half were injected anti-OX40 mAb in addition to the M25_409–423_ SLP. **(B)** The total size of the splenic M25_409–423_ SLP vaccine-induced CD4^+^ T cells from each group was measured by intracellular cytokine staining. Representative plots depict percentages of IFN-γ versus TNF cytokine producing CD4^+^ T cell populations at day 8 post-booster vaccination. **(C)** Total numbers of splenic IFN-γ^+^ producing M25_409–423_ antigen-specific CD4^+^ T cells at day 8 post booster SLP vaccination and differential anti-OX40 mAb treatment are shown. **(D)** Total double (IFN-γ/TNF) and **(E)** triple (IFN-γ/TNF/IL-2) cytokine producers of M25_409–423_ vaccine-specific CD4^+^ T cells measured in spleen at day 8 post-booster vaccination. Fold differences among each population are also depicted **(F)**. Pie charts show the percentages of the single (IFN-γ), double (IFN-γ/TNF), and triple (IFN-γ/TNF/IL-2) cytokine producers of each M25_409–423_-specific CD4^+^ T cell population upon vaccination with M25_409–423_ SLP and anti-OX40 mAb. Data represent mean values and are representative of three independent experiments (*n* = 5–6 per group). *P < 0.05; **P < 0.01; ns, not significant.

Next, we attempted to augment the magnitude of the SLP-induced CD4^+^ T cell responses. As OX40-mediated signals are important for enhancing CD4^+^ T cell expansion and survival (28), we decided to use an agonistic OX40 antibody that provides *in vivo* OX40 stimulation. First, we investigated the scheduling of the agonistic OX40 antibody administration (i.e., during priming only, during booster only or during priming and booster) in order to obtain the most optimal CD4^+^ T cell stimulation (Figure 2A). The magnitude of the T cell response elicited by the SLP containing the M25_409–423_ epitope was measured 8 days post-booster vaccination in the spleen. OX40 stimulation clearly increased the magnitude of the M25_409–423_-specific CD4^+^ T cell responses, and remarkably, this was most prominent when the mice received agonistic OX40 antibody during the booster vaccination only (Figures 2B,C). Markedly, a >100-fold increase in IFN-γ^+^ CD4^+^ T cells was observed when compared to SLP vaccination without enforced OX40 stimulation, whereas the response was 17-fold and 5-fold higher than in mice receiving OX40 antibody during priming only or during both priming and booster vaccination, respectively (Figure 2C). In addition, there was a striking gain in cytokine polyfunctionality when agonistic OX40 antibody was provided during booster vaccination only (Figures 2D–F). Compared to SLP vaccination, the increase in absolute numbers of triple IFN-γ/TNF/IL-2 producers was even >200-fold (Figure 2E).

**Figure 3:**
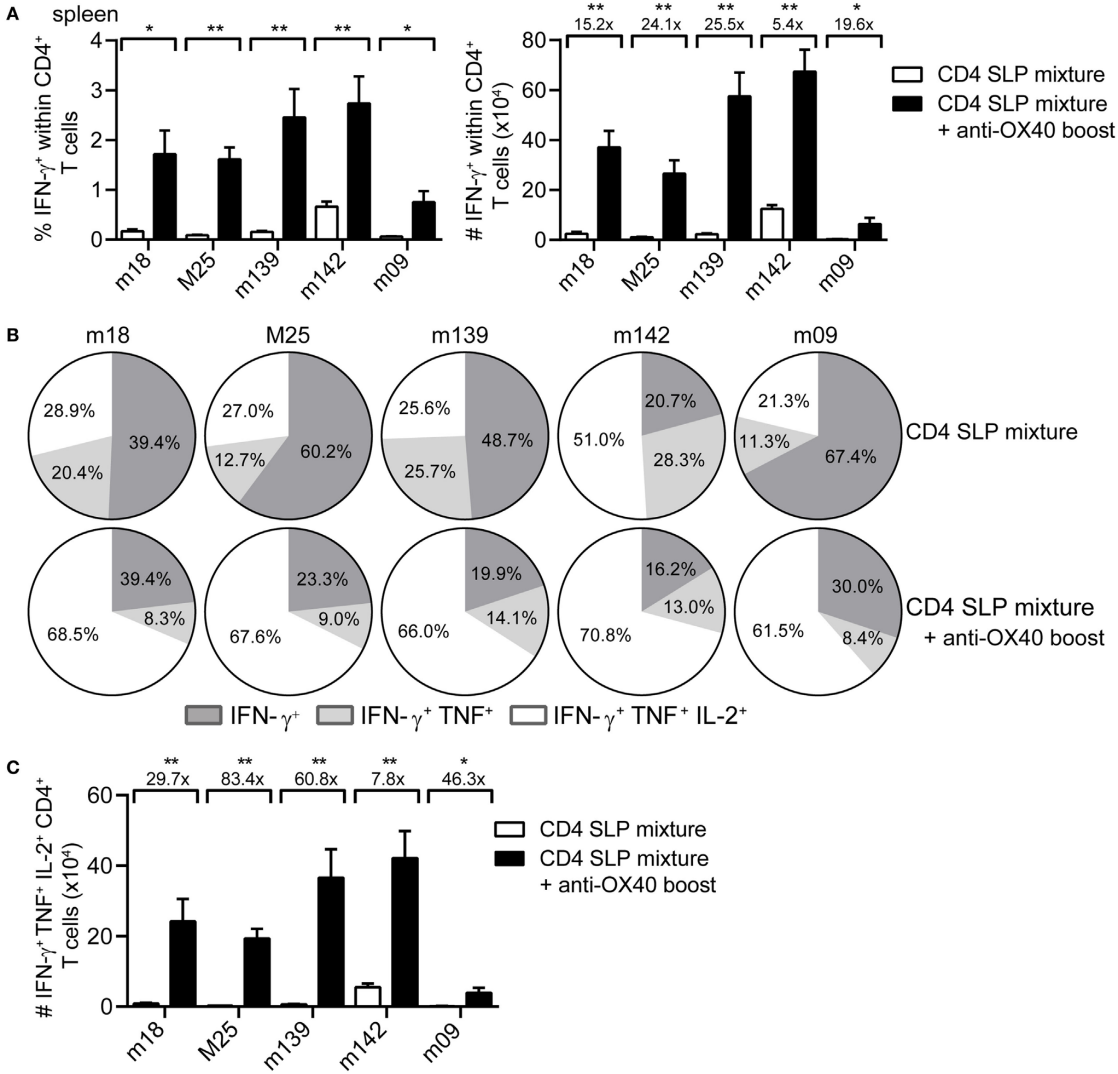
**OX40 activation during booster vaccination with combinatorial MHC class II synthetic long peptide (SLP) vaccines leads to the induction of robust and polyfunctional CD4^+^ T cell responses**. **(A)** Percentages (left) and total numbers (right) of the splenic epitope-specific IFN-γ^+^ CD4^+^ T cell responses elicited at day 8 post-booster vaccination with a mixture of SLPs containing MHC class II epitopes alone (CD4 SLP mixture; white bars) or with SLPs containing MHC class II epitopes and anti-OX40 mAb (CD4 SLP mixture + anti-OX40 boost; black bars). **(B)** Pie charts show the percentages of the single (IFN-γ), double (IFN-γ/TNF), and triple (IFN-γ/TNF/IL-2) cytokine producers of each antigen-specific CD4^+^ T cell population at day 8 post-booster vaccination with a mixture of MHC class II SLPs alone or with MHC class II SLPs plus anti-OX40 mAb. **(C)** Total triple (IFN-γ/TNF/IL-2) cytokine producers of each antigen-specific CD4^+^ T cell population were measured in spleen at day 8 post-booster vaccination with a mixture of MHC class II SLPs and anti-OX40 mAb or with SLPs alone. Fold difference is indicated. Data represent mean values and are representative of three independent experiments (*n* = 6 per group). *P < 0.05; **P < 0.01.

Next, we examined whether the strong increase in MCMV-specific CD4^+^ T cells by administration of agonistic OX40 antibody during booster vaccination was also evident in case of a mixture of SLPs. Clearly, at day 8 post-booster vaccination, a strong increase in both percentages and absolute numbers of the peptide-specific IFN-γ^+^ CD4^+^ T cells was observed for all epitopes in the mixture (Figure 3A). In addition, administration of OX40 antibody dramatically improved the cytokine polyfunctional traits (Figure 3B). Most profoundly, OX40 stimulation augmented the IL-2 production capacity of the vaccine-induced CD4^+^ T cells (Figures 3B,C). Together, these results demonstrate that activation of the OX40 axis during booster SLP vaccination leads to superior CD4^+^ T cell expansion and induction of cytokine polyfunctionality.

Having established a powerful means to augment SLP vaccines containing MHC class II epitopes, we tested if the used SLPs may comprise unidentified class I epitopes and/or linear B cell epitopes leading to CD8^+^ T cells and antibody responses, respectively. However, intracellular cytokine staining did not reveal any induction of MCMV-specific CD8^+^ T cells and SLP-specific antibody ELISAs were negative (Figures S1A,B in Supplementary Material). Furthermore, increased percentages of activated NK cells were also not detected after SLP vaccination (Figure S1C in Supplementary Material), indicating that the intended MHC class II epitope-containing SLPs with enforced OX40 stimulation exclusively activate antigen-specific CD4^+^ T cell responses.

### Provision of OX40 Stimulation during Booster Vaccination Also Advances SLP-Induced CD8^+^ T Cell Responses

To improve our previously reported CD8^+^ T cell eliciting SLP vaccine modality (22), we here envisaged to combine both MHC class I and II epitope-containing SLPs. We, therefore, also analyzed the impact of OX40 engagement on vaccine-induced CD8^+^ T cells in a similar scheduling experiment using a SLP exclusively containing the CD8^+^ T cell peptide epitope M57_816–824_ (Figure S2A in Supplementary Material). Consistent with the results described for CD4^+^ T cells, mice that were vaccinated and treated with agonistic OX40 antibody during booster vaccination displayed the strongest SLP-induced CD8^+^ T cell response in both blood and spleen (Figures S2B–F in Supplementary Material). The number of SLP-induced CD8^+^ T cells, determined either by IFN-γ reactivity or by MHC class I tetramer binding, at the peak after the boost were similar, indicating that OX40 stimulation during SLP vaccination (provided either during priming, during booster or during prime/boost) induces functional (non-exhausted) CD8^+^ T cells (Figures S2C,D in Supplementary Material). The cell-surface phenotype based on KLRG1 and CD127 of the vaccine-induced CD8^+^ T cells at the peak of the response after the booster was comparable (Figure S2G in Supplementary Material).

**Figure 4:**
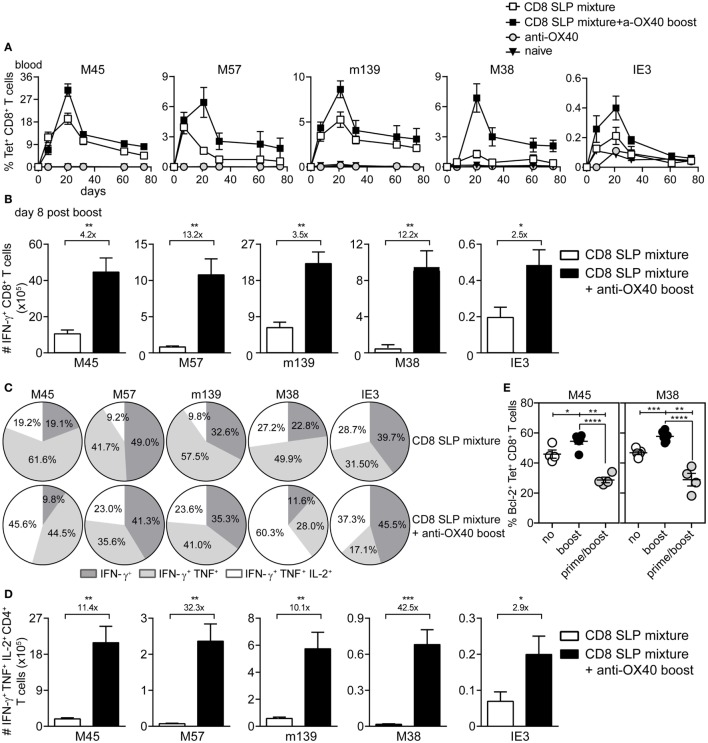
**OX40 ligation during booster vaccination with a mixture of MHC class I epitope-containing synthetic long peptides (SLPs) leads to the induction of strong and polyfunctional CD8^+^ T cell responses**. **(A)** Longitudinal analysis of the epitope-specific CD8^+^ T cell responses in blood induced by combinatorial MHC class I epitope-containing SLP vaccines without (CD8 SLP mixture) and with anti-OX40 mAb administration (given only during booster vaccination) (CD8 SLP mixture + a-OX40 boost). Data represent mean values ± SEM (*n* = 12 per group). **(B)** Total IFN-γ^+^ cytokine-producing CD8^+^ T cells for each antigen-specific population detected in spleen at day 8 post-booster vaccination with CD8 SLP mixture (white bars) or with CD8 SLP mixture + anti-OX40 boost (black bars). **(C)** Pie charts show the percentages of the single (IFN-γ), double (IFN-γ/TNF), and triple (IFN-γ/TNF/IL-2)-specific cytokine producers of the antigen-specific CD8^+^ T cell populations at day 8 post-booster vaccination. **(D)** Total numbers of IFN-γ/TNF/L-2 cytokine producing CD8^+^ T cells for each antigen-specific population detected in spleen at day 8 post-booster vaccination. **(E)** At day 8 after booster vaccination with combined MHC class I SLPs and/or anti-OX40 mAb (during booster or during both prime/boost), the BCL-2 protein expression was measured within the antigen-specific CD8^+^ T cells in spleen by flow cytometry. Fold changes between groups are depicted. Data represent mean values + SEM (*n* = 6 mice per group) and are representative of three independent experiments. *P < 0.05; **P < 0.01; ***P < 0.001; ****P < 0.0001.

Next, the impact of OX40 stimulation during booster vaccination was evaluated for a mixture of MHC class I epitope-containing SLPs. Longitudinal analysis of the SLP-specific CD8^+^ T cell response revealed that OX40 stimulation during booster vaccination clearly amplifies the expansion of all SLP vaccine-induced CD8^+^ T cells measured in blood and spleen (Figures 4A,B), although this effect was relatively lower when compared to that seen for CD4^+^ T cells. At the peak of the response, 8 days after booster SLP vaccination, the magnitude of the IFN-γ^+^CD8^+^ T cell response specific for each MHC class I-restricted epitope was again proportional to the MHC class I tetramer response (data not shown). However, the polyfunctional cytokine profile was improved upon OX40 stimulation (Figures 4C,D). In particular, OX40 stimulation augmented the percentages and absolute number of the triple cytokine producers (Figures 4C,D).

To gain insight into the mechanisms underlying the apparent impact of OX40 stimulation during booster vaccination, we examined the expression of the pro-apoptotic protein BCL-2, a known target of OX40 triggering and implicated in T cell survival (29). BCL-2 expression was upregulated by the vaccine-specific CD8^+^ T cells when OX40 stimulation was provided during booster vaccination compared to no OX40 stimulation (Figure 4E). Moreover, when agonistic OX40 antibody was administered during both primary and booster vaccination, BCL-2 expression was downregulated. These results indicate that stimulation of OX40 can bolster vaccine-induced CD8^+^ T cell expansion through a BCL-2-dependent mechanism, if this stimulation is correctly scheduled.

**Figure 5:**
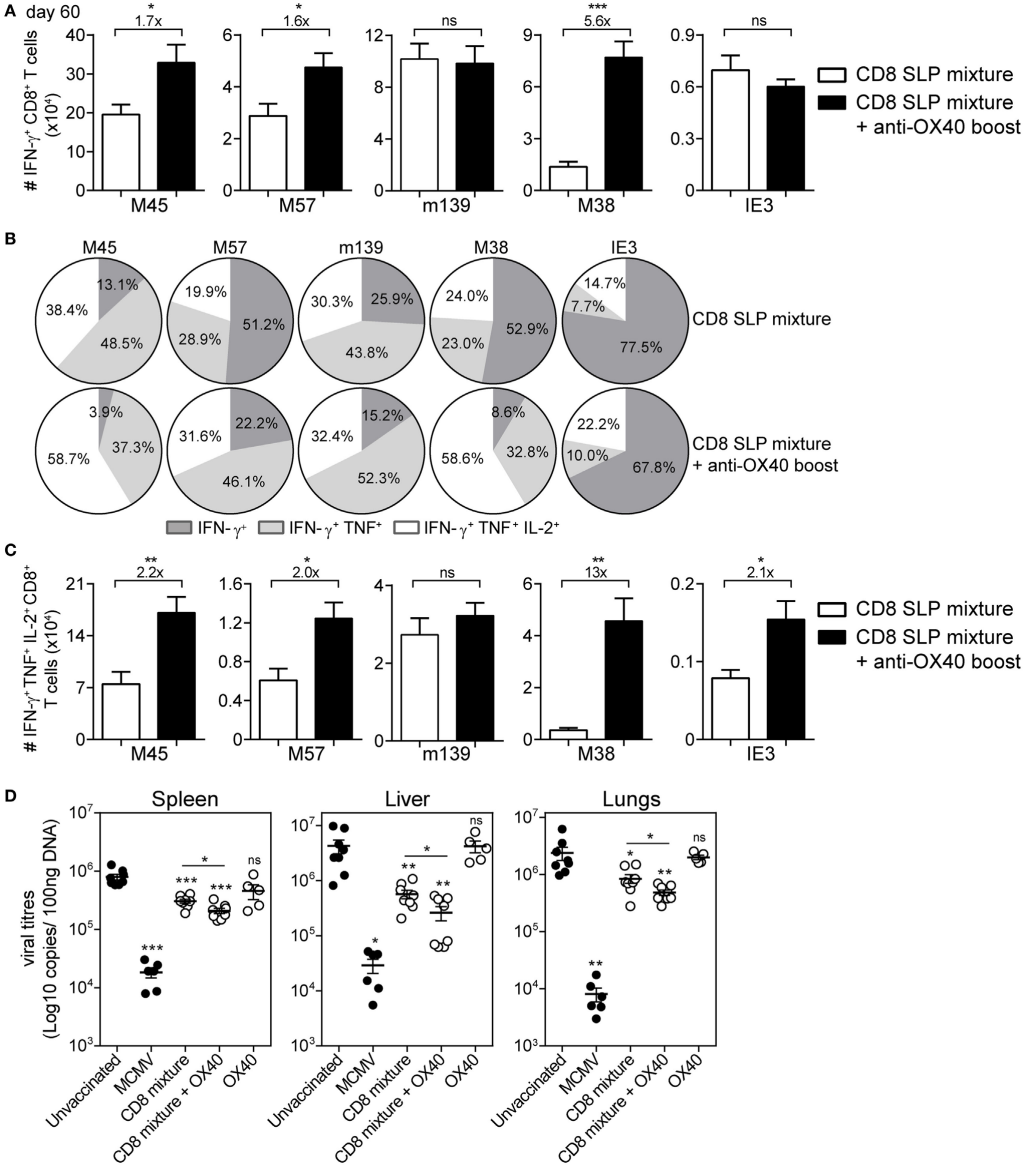
**Agonistic OX40 mAb administration during booster vaccination leads to improved memory synthetic long peptide (SLP)-elicited CD8^+^ T cell responses**. **(A)** Total single IFN-γ^+^ cytokine-producing CD8^+^ T cells for each antigen-specific population detected in spleen at day 60 post-booster vaccination with combinatorial MHC class I SLPs and anti-OX40 mAb (provided during booster vaccination) (CD8 SLP mixture + anti-OX40 boost) or with MHC class I SLPs alone (CD8 SLP mixture). Fold changes between groups are depicted. Data represent mean values + SEM (*n* = 6 per group). **(B)** Pie charts show the percentages of the single (IFN-γ), double (IFN-γ/TNF), and triple (IFN-γ/TNF/IL-2) cytokine producers of each antigen-specific CD8^+^ T cell population in spleen at day 60 post-booster vaccination with a mixture of MHC class I SLPs and anti-OX40 mAb (administered only during booster vaccination) or with MHC class I SLPs alone. **(C)** Total triple IFN-γ^+^/TNF^+^/IL-2^+^-producing CD8^+^ T cells for each antigen-specific population detected in spleen at day 60 post-booster vaccination. Fold changes between groups are depicted. Data represent mean values + SEM (*n* = 6 per group). **(D)** Different groups of mice [unvaccinated (naive), mouse CMV (MCMV) infected (live-virus vaccine), vaccinated with a mixture of all 5 MHC class I SLPs, vaccinated with a mixture of all five MHC class I SLPs and anti-OX40 mAb (given only during booster vaccination), or treated with a-OX40 mAb only] were challenged at day 60 postvaccination/infection with 5 × 10^4^ PFU salivary gland-derived MCMV Smith. At day 5, post challenge spleen, liver, and lungs were harvested and the viral genome copies were determined by qPCR. The viral titers of individual mice are depicted (*n* = 5–8 mice per group). Mean ± SEM is indicated. The detection limit was below 1,000 genome copies as measured in naive mice. Experiments were performed twice with similar outcome. *P < 0.05; **P < 0.01; ***P < 0.001; ns, not significant as compared to the unvaccinated group unless otherwise indicated.

Over time, the SLP-induced CD8^+^ T cell responses contracted, yet the OX40 boosted epitope-specific CD8^+^ T cell responses to M38, M45, and M57 were maintained at higher levels (Figure 5A). At the memory phase (60 days after booster vaccination), there were higher percentages and numbers of triple (IFN-γ/TNF/IL-2) cytokine-producing vaccine-induced CD8^+^ T cells in mice that received agonistic OX40 antibody during booster vaccination (except for m139_419–426_) (Figures 5B,C).

We also tested the impact of the *in vivo* OX40 stimulation on the secondary expansion potential, a hallmark of memory T cells. We performed adoptive transfer experiments in which congenically marked (CD45.1^+^) memory CD8^+^ T cells from SLP vaccinated mice were isolated and transferred into naïve recipient mice, which were subsequently challenged with MCMV (Figure S3A in Supplementary Material). Overall, the SLP-induced memory CD8^+^ T cells isolated from mice that also received OX40 stimulation during booster vaccination expanded better compared to the vaccine only group (Figures S3B,C in Supplementary Material).

Subsequently, we examined whether OX40 stimulation is able to further improve the prophylactic efficacy of the combined MHC class I SLP vaccine. At day 60, post-booster vaccination, mice were virally challenged and the titers were quantified in spleen, liver, and lungs. Similar to our previous study (22), vaccination with a mixture of MHC class I SLPs resulted in reduction of the virus titers (Figure 5D). Mice that received OX40 stimulation during the booster showed increased potency to control the virus compared to mice that received no extra stimulation of OX40 (Figure 5D). All together, these data suggest that enforced OX40 stimulation during booster SLP vaccination does not only impact the expansion of activated CD8^+^ T cells but also has a long-lasting constructive influence on the magnitude and the functional profile of the vaccine-elicited CD8^+^ T cells leading to more effective viral control.

### SLP Vaccines Inducing Both CD4^+^ and CD8^+^ T Cells Confer Superior Protection against MCMV Infection

**Figure 6:**
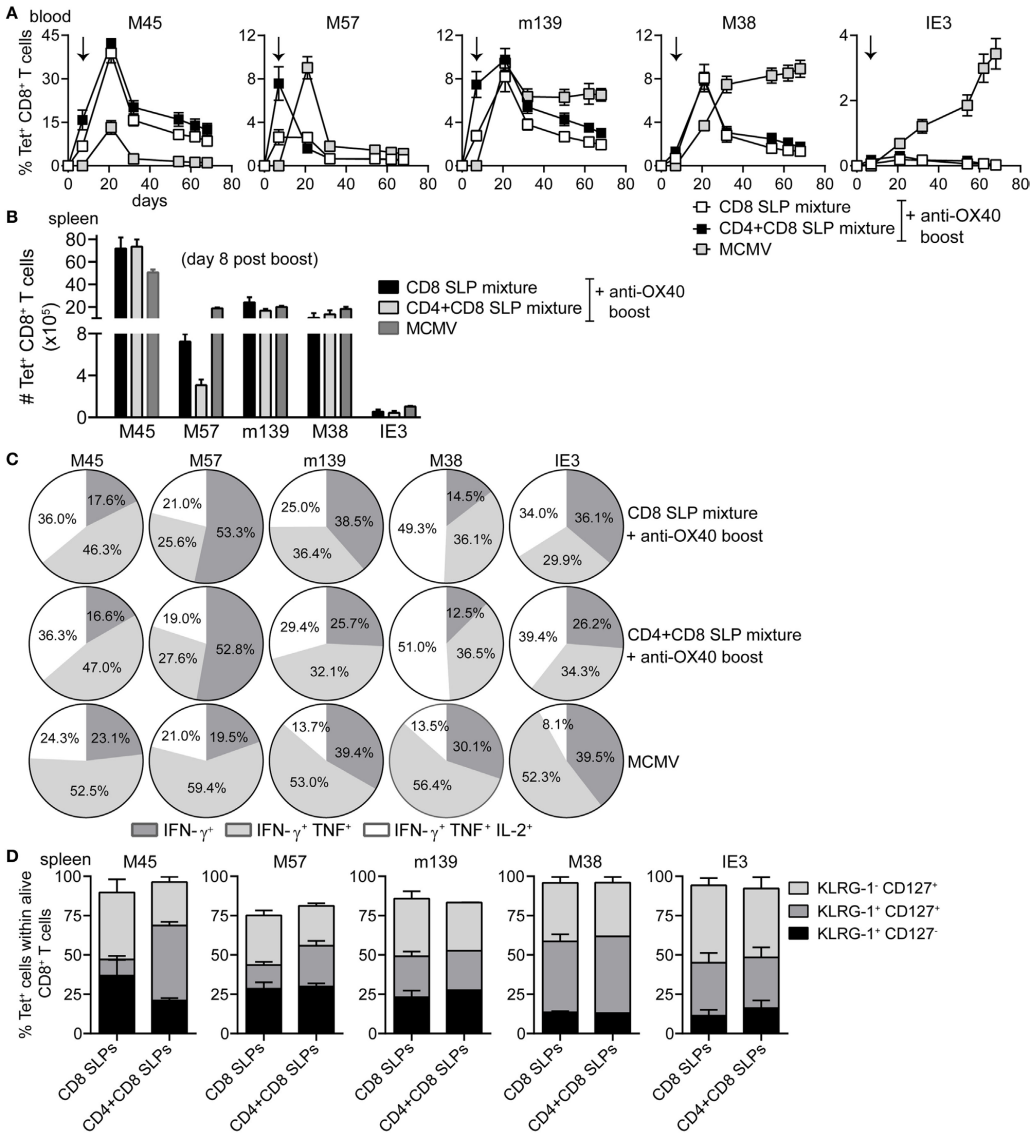
**Combination of MHC class I and II epitope-containing synthetic long peptide (SLP) vaccines and OX40 agonistic mAb during booster vaccination leads to robust induction of both CD4^+^ and CD8^+^ T cell responses**. C57BL/6 mice were vaccinated s.c. with a combination of all MHC class I epitope-containing SLPs (CD8 SLP) or with a combination of all MHC class I and II epitope-containing SLPs (CD4 + CD8 SLP). In both groups agonistic OX40 mAb was administered i.p. during booster vaccination. **(A)** Kinetics of the antigen-specific CD8^+^ T cells measured by MHC class I tetramer staining in blood. Data shown are mean values ± SEM (*n* = 18 mice/group) **(B)** Total MHC class I tetramer-specific CD8^+^ T cells induced by combinatorial MHC class I or MHC class I plus MHC class II SLP vaccination compared to MCMV infection at day 8 post-booster vaccination in spleen. Data represent mean values (*n* = 5 mice/group). **(C)** The cytokine production capacity of the splenic SLP vaccine-induced CD8^+^ T cells was examined by intracellular cytokine staining at day 8 after booster vaccination. Pie charts depict the percentages of the single (IFN-γ), double (IFN-γ/TNF), and triple (IFN-γ/TNF/IL-2) cytokine producers of each antigen-specific CD8^+^ T cell populations at day 8 post-booster vaccination. **(D)** Phenotypic analysis of the combinatorial SLP vaccine-induced CD8^+^ T cells in spleen at day 8 post-booster vaccination. Data represent mean values (*n* = 5 mice/group) and are representative of three independent experiments.

To evaluate if the provision of CD4^+^ T cells would benefit the CD8^+^ T cell response, C57BL/6 mice were vaccinated with a mixture of either all 5 MHC class I epitope-containing SLPs or with a mixture of all 5 MHC class I and all 5 class II epitope-containing SLPs (Table 1 in Supplementary Material). Agonistic OX40 mAb was provided during the boost only. The CD8^+^ T cell response after the prime vaccination was higher when the SLP vaccine contained a mixture of MHC class I and II peptide epitopes (Figure 6A; Figure S4A in Supplementary Material), stressing the importance of CD4^+^ T cell help during the priming. Also, the phenotype of the vaccine-induced CD8^+^ T cells after prime vaccination revealed slightly more effector-type (KLRG-1^+^CD127^-^) CD8^+^ T cells when CD4^+^ T cell help was provided (Figure S4B Supplementary Material), whereas the polyfunctionality of the vaccine-induced CD8^+^ T cells remained unchanged (data not shown). However, at day 7/8 after booster SLP vaccination, the effect of CD4^+^ T cell help on the magnitude of the vaccine-induced CD8^+^ T cell response was no longer detectable in blood (Figure 6A) and spleen (Figure 6B). At this time-point, also no difference in the CD8^+^ T cell cytokine polyfunctionality and phenotype was found (Figures 6C,D). Furthermore, the size and polyfunctionality of the CD4^+^ T cells responses induced at the peak response after combined MHC class I and II SLP vaccination was similar to vaccination with MHC class II SLPs only (Figures S5A,B in Supplementary Material). Taken together, vaccination with a mixture of MHC class I and II epitope-containing SLPs resulted in enhanced primary CD8^+^ T cell expansion compared to vaccination with class I epitope-containing SLPs only, but no additional effects of the helper T cells were observed after the enforced OX40 stimulation provided during booster vaccination.

**Figure 7:**
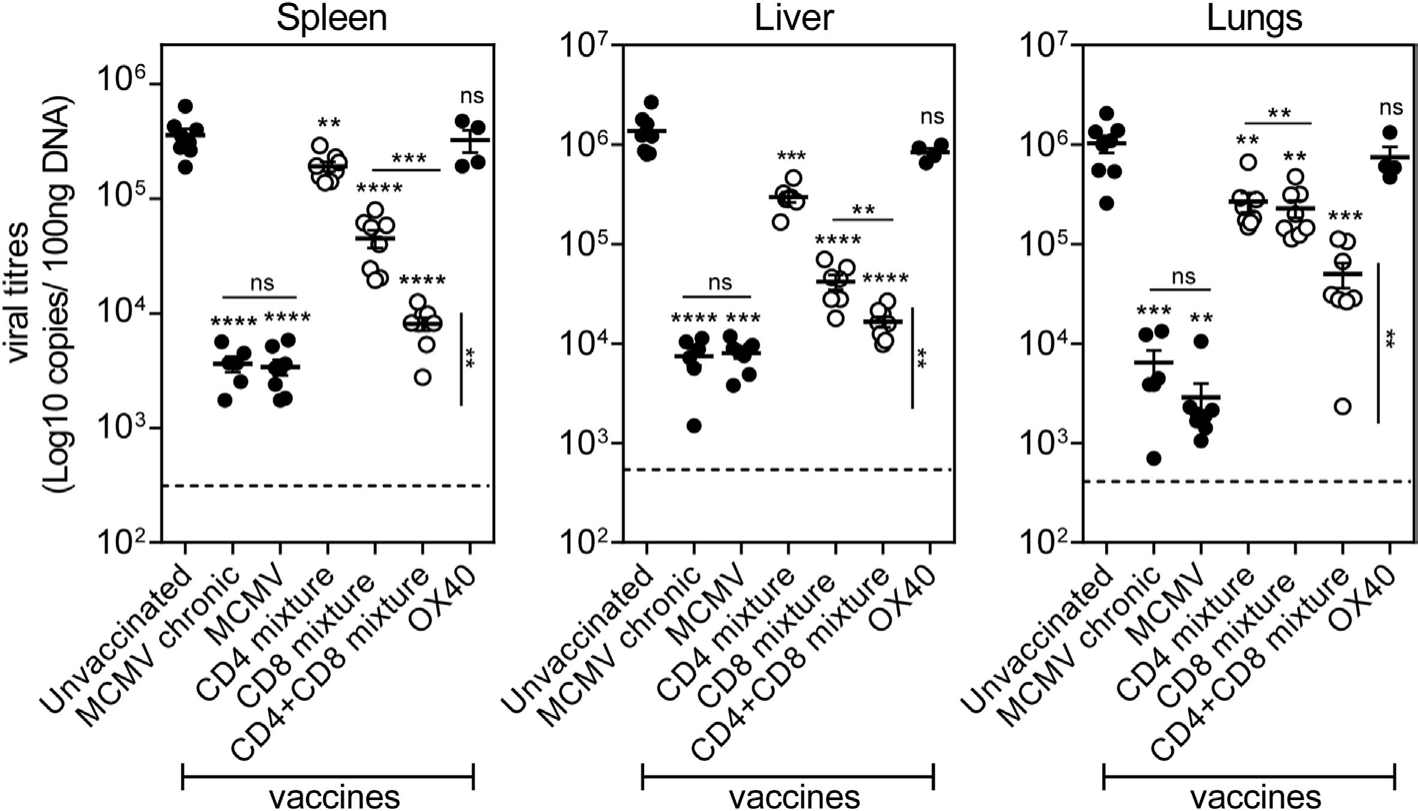
**Prime-boost vaccination with a combination of MHC class I and II epitope-containing synthetic long peptides (SLPs) exhibits increased potency to protect against lytic mouse CMV (MCMV) infection**. Unvaccinated (naive), MCMV (live-virus vaccine), combined MHC class II SLPs (CD4 mixture), combined MHC class I SLPs (CD8 mixture), combined MHC class I and II SLPs (CD4 + CD8 mixture), and anti-OX40 mAb only treated C57BL/6 mice were challenged 60 days post-booster vaccination/infection with 5 × 10^4^ PFU salivary gland-derived MCMV Smith. At day 5 post challenge, liver, lungs, and spleen were harvested and the viral genome copies were quantified by qPCR. To further evaluate the efficacy of the vaccines, the viral titers of chronically infected (MCMV chronic/day 60) mice were also measured. The viral titers of individual mice are depicted (*n* = 4–8 per group). Statistical significance between the unvaccinated group and the rest of the groups is indicated with asterisks above each group. A statistical comparison between the CD4 + CD8 mixture group and the MCMV (live-virus vaccine) and MCMV chronic group was also performed (vertical statistical bar). For both comparisons the statistical difference was found the same (**P < 0.01). The detection limit was below 1,000 genome copies as measured in naive mice. Experiment was performed twice with similar outcome. Statistical difference is indicated **P < 0.01; ***P < 0.001; ****P < 0.0001; ns, not significant as compared to the unvaccinated group unless otherwise indicated.

Ultimately, we analyzed the prophylactic efficacy of the SLP-induced MCMV-specific CD4^+^ and CD8^+^ T cells to control viral replication. At day 60 post-booster SLP vaccination, mice were infected with MCMV and 5 days later the viral titers were quantified. The viral load of unvaccinated (naive) mice challenged with MCMV was found significantly higher in spleen, liver, and lungs compared to the viral load of mice that received earlier a virulent virus as a vaccine. This result suggests that pre-existing immunity to MCMV can inhibit virus replication during subsequent infection (Figure 7). Furthermore, the viral titers of mice that had received the virulent virus-based vaccine were similar to those measured during chronic MCMV infection, indicating that this is the maximum of immune control that can be achieved. Mice vaccinated with the mixture of SLPs containing MHC class II epitopes plus OX40 triggering during booster vaccination displayed significant reduction in viral load in all tested organs (Figure 7), indicating that the SLP-induced MCMV-specific CD4^+^ T cells display direct antiviral properties. Vaccination with the mixture of MHC class I SLPs plus anti-OX40 during booster resulted in a more efficient reduction of viral load in all organs. Strikingly, mice that received a mixture of MHC class I and II epitope-containing SLPs and OX40 stimulation during booster vaccination displayed the strongest reduction in viral load (Figure 7). Notably, the SLP vaccine-induced reduction in viral load in the spleen and liver was almost as effective as to what is observed after vaccination with virulent virus. Thus, SLP vaccines comprising a mixture of MHC class I and II SLPs has the highest protection potency compared to similar vaccines that elicit merely MCMV-specific CD4^+^ or CD8^+^ T cell responses. All together, we conclude that OX40 activation during booster vaccination empowers SLP-induced memory CD4^+^ and CD8^+^ T cells to efficiently counteract lytic MCMV infection.

## Discussion

In this study, we show that SLP-based vaccine strategies eliciting vigorous polyfunctional CD4^+^ and CD8^+^ T cell responses are highly effective against MCMV infection. While SLP-based vaccines that evoke solely CD8^+^ T cell responses display already efficacy against lytic MCMV infection (22), vaccination with a mixture of various immunodominant MHC class II and MHC class I MCMV epitopes and the combination with enforced OX40 stimulation results in superior vaccine efficacy. Former explored CMV vaccines focused mainly on the induction of neutralizing antibodies and thus far did not show substantial efficacy (3–5). Our finding that SLP-based vaccines that merely provoke CD4^+^ and CD8^+^ T cell responses are almost as efficient as a virulent vaccine suggests that the inclusion of T cell-stimulating antigens should facilitate the design of more efficient vaccines against CMV.

Signals through the OX40 costimulatory receptor are known to regulate expansion and survival of both CD4^+^ and CD8^+^ T cells after antigen encounter (28, 30). Consistent with this, OX40 had a robust effect on the magnitude of the effector SLP vaccine-induced CD4^+^ and CD8^+^ T cells and on their capacity to induce “Th1” cytokine responses (especially IL-2). This effect of OX40 costimulatory signals was stronger when agonistic antibody to OX40 was provided during booster SLP vaccination. We hypothesize that primed T cells more rapidly and stronger upregulated OX40 leading to greater benefit during enforced OX40 stimulation. OX40 ligation seems to work better on CD4^+^ T cells as compared to CD8^+^ T cells, which may be related to the higher expression of OX40 on CD4^+^ T cells (31, 32). Due to its capacity to regulate both CD4^+^ and CD8^+^ T cells, OX40 is a promising candidate in immunotherapy of chronic viral infections and cancer (30). In this study, we did not detect toxicity of OX40 agonistic antibodies but a better understanding of potential side-effects is required.

CD4^+^ T cell responses in CMV infection have been long known as important contributors to control primary infection (8, 33). In MCMV infection, CD4^+^ T cells direct the quality and persistence of inflationary CD8^+^ T cells and B cell responses (23, 24, 34). Here, we show that vaccine-induced CD4^+^ T cells solely can confer moderate protection against acute MCMV infection. These results are consistent with other reports showing CD4^+^ T cell effectivity against MCMV and HCMV (8, 12, 25, 35–37). Furthermore, we observed that addition of CD4^+^ T cell help during vaccination with MHC class I SLP vaccines promoted priming of naïve CD8^+^ T cells. After booster vaccination in settings wherein OX40 costimulation was enhanced, no additional effects of CD4 help on the magnitude and phenotype of the CD8^+^ T cells were observed, suggesting that enforced OX40 stimulation during booster vaccination may replace the need for CD4 help signals. On this basis, the improved prophylactic vaccine efficacy of the combined MHC class I and II SLP vaccines appears to be more additive rather than synergistic.

Another interesting observation was that the vaccine efficacy was somewhat better in liver and spleen than in lungs. A possible explanation for this discrepancy is that the SLP-induced CD8^+^ T cells are better capable to control the virus (lowering the viral titers) in the spleen and liver than the SLP-induced CD4^+^ T cells. A marked difference in the efficacy of SLP-induced CD4^+^ and CD8^+^ T cells as observed in liver and spleen is not found in the lungs and can thus be dictated by the tissue environment. Differences in site-specific control of MCMV-specific CD4^+^ and CD8^+^ T cells is known to be especially important in the salivary glands but for other tissues this may be important as well (33). Nevertheless, the combined SLP vaccines inducing both CD4^+^ and CD8^+^ T cell responses clearly improved the efficacy of the vaccine in all organ tissues. In this respect, it is of interest to note that SLP-based vaccines can be further refined by different prime-booster regimens, inclusion of B cell epitopes and by combinations with adjuvants, immunomodulatory antibodies, or other vaccine platforms (38).

This study provided evidence that SLP-based vaccines eliciting broad CD4^+^ and CD8^+^ T cell responses can effectively control lytic MCMV infection without contribution by humoral responses. The use of OX40 as an adjuvant for MCMV peptide immunization strongly bolstered the development of effective CD4^+^ and CD8^+^ T cells. Future studies to examine the ability of the SLP vaccine-induced T cells and OX40 costimulation to boost immune responses in immunocompromised settings of CMV infection or other chronic viral infections are strongly encouraged by these promising findings. Taken together, our data highlight the importance of designing CMV vaccines that elicit effective CD4^+^ and CD8^+^ T cell responses.

## Author Contributions

EP, RA, and SB designed research. EP and RA performed the experiments and analyzed the data. LB contributed essential reagents. EP, RA, and SB wrote the manuscript.

## Conflict of Interest Statement

This study has been conducted by the Leiden University Medical Center (LUMC) that holds a patent on the synthetic long peptides as vaccine (US 7.202.034). SB is named as inventor on this patent. LB is a shareholder of Bioceros that holds a patent on anti-human OX40 mAbs. The other authors declare no conflict of interest.
