# Supplementary material for: Enforced OX40 Stimulation Empowers Booster Vaccines to Induce Effective CD4+ and CD8+ T Cell Responses against Mouse Cytomegalovirus Infection
Source: Front Immunol. 2017 Feb 20;8:144. doi: 10.3389/fimmu.2017.00144 (PMC5316540; doi:10.3389/fimmu.2017.00144)
Supplement: Supplementary file 1 [file table_1.pdf]

Supplementary Table 1. Depiction of the MHC class I and II peptide epitopes and SLPs used in this study.

Mouse strain	**Protein (ORF)**	SLP sequence *	Epitope residues (aa)	MHC allele - restriction	**Stage of expression**	Inflation?	**Human CMV Homologue**
C57BL/6	m18	**NERAKSPAAMTAEDE**	872–886	I-A^{b}	Immediate early gene	No	
C57BL/6	M25	**NHLYETPISATAMVI**	409–423	I-A^{b}	UL25 family homologue, tegument protein	No	UL25 (GF1)
C57BL/6	m139	**TRPYRYPRVCDASLS**	560–574	I-A^{b}	US22 family homologue	No	US22
C57BL/6	m142	**RSRYLTAAAVTAVLQ**	24–38	I-A^{b}	US22 family homologue, with m143 block of PKR activation	No	US26
C57BL/6	m09	**GYLYIYPSAGNSFDL**	133–147	I-A^{b}	Glycoprotein family, nonessential	Yes	
C57BL/6	M45	REDVVK**HGIRNASFI**TGCSA	985-993	H-2-D^{b}	Early gene	No	UL45
C57BL/6	M57	FPACGL**SCLEFWQRV**LQNS	816-824	H-2-K^{b}	Early gene	No	UL57
C57BL/6	m139	VVLVGARG**TVYGFCLL**SND	419-426	H-2-K^{b}	Early gene	Yes	US22
C57BL/6	M38	VTLI**SSPPMFRV**PVNPVPGG	316-323	H-2K^{b}	Early gene	Yes	UL38
C57BL/6	IE3	DKSRKYPA**RALEYKNL**PFR	416-423	H-2-K^{b}	Immediate early gene	Yes	IE2

* Bold underlined amino acid (aa) residues within the SLP sequence indicate the CTL epitope.
