# Supplementary figures and images for: Enforced OX40 Stimulation Empowers Booster Vaccines to Induce Effective CD4+ and CD8+ T Cell Responses against Mouse Cytomegalovirus Infection
Source: Front Immunol. 2017 Feb 20;8:144. doi: 10.3389/fimmu.2017.00144 (PMC5316540; doi:10.3389/fimmu.2017.00144)

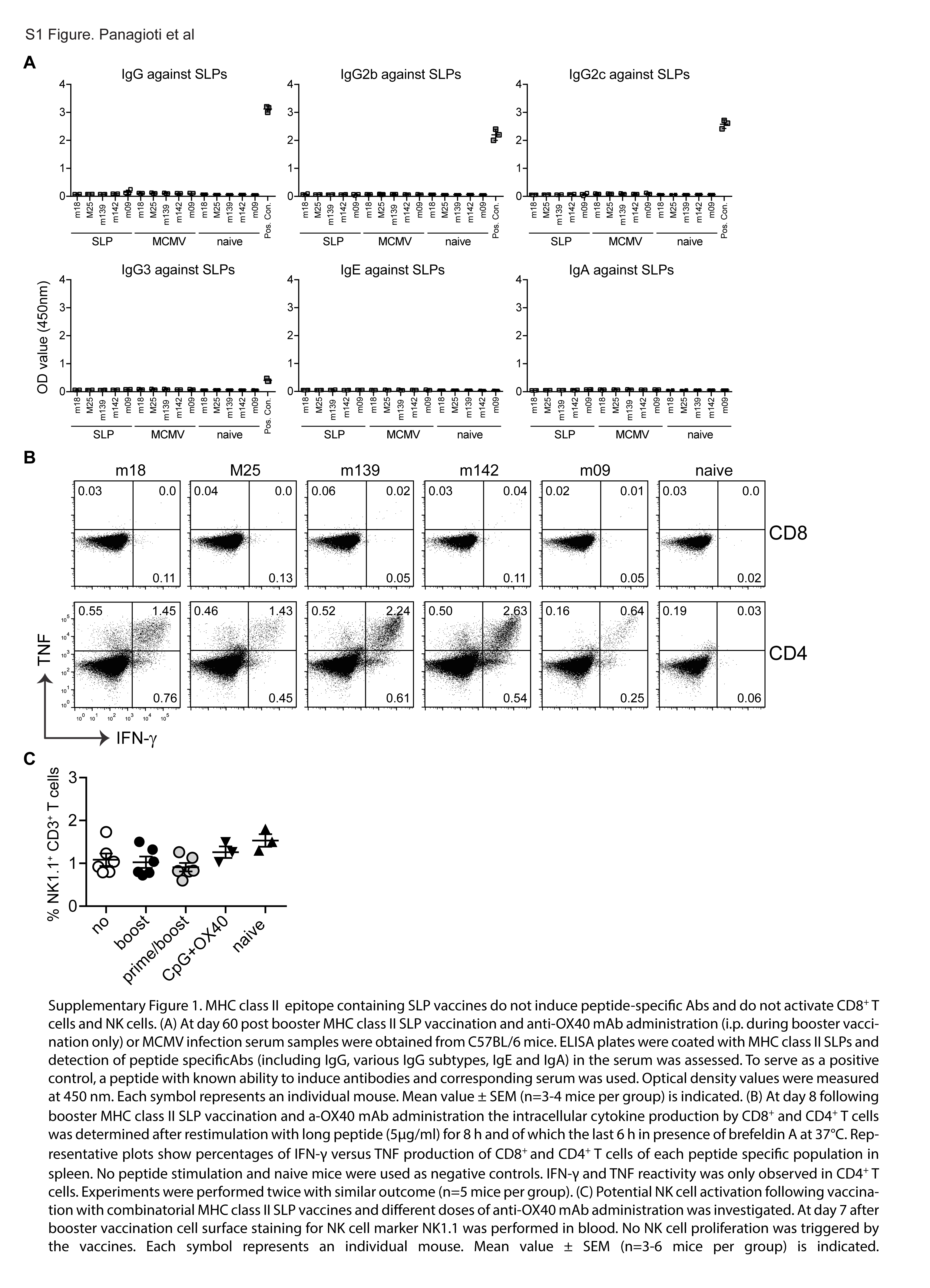

Supplement: Supplementary file 2 [file image_1.tif]

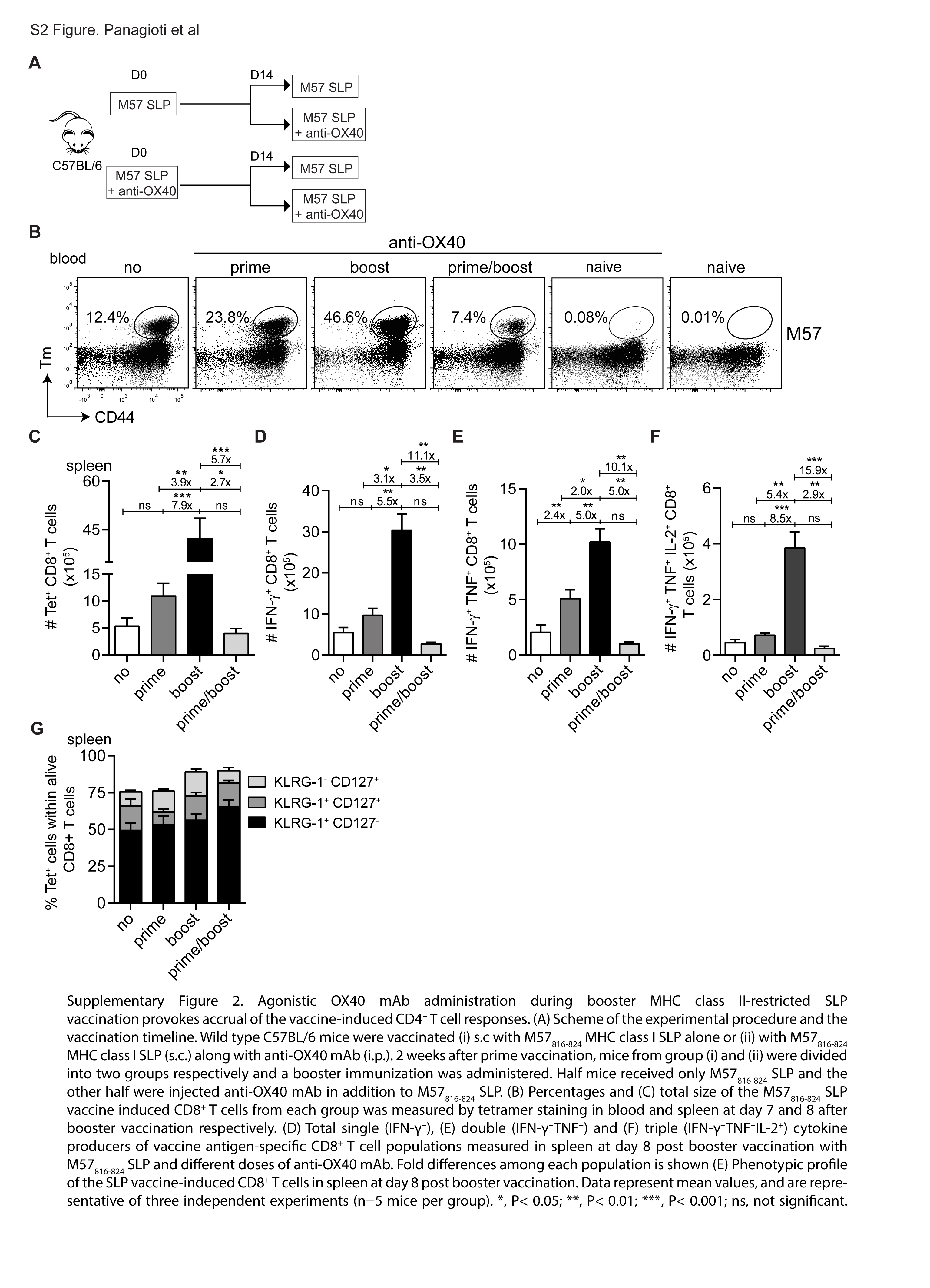

Supplement: Supplementary file 3 [file image_2.tif]

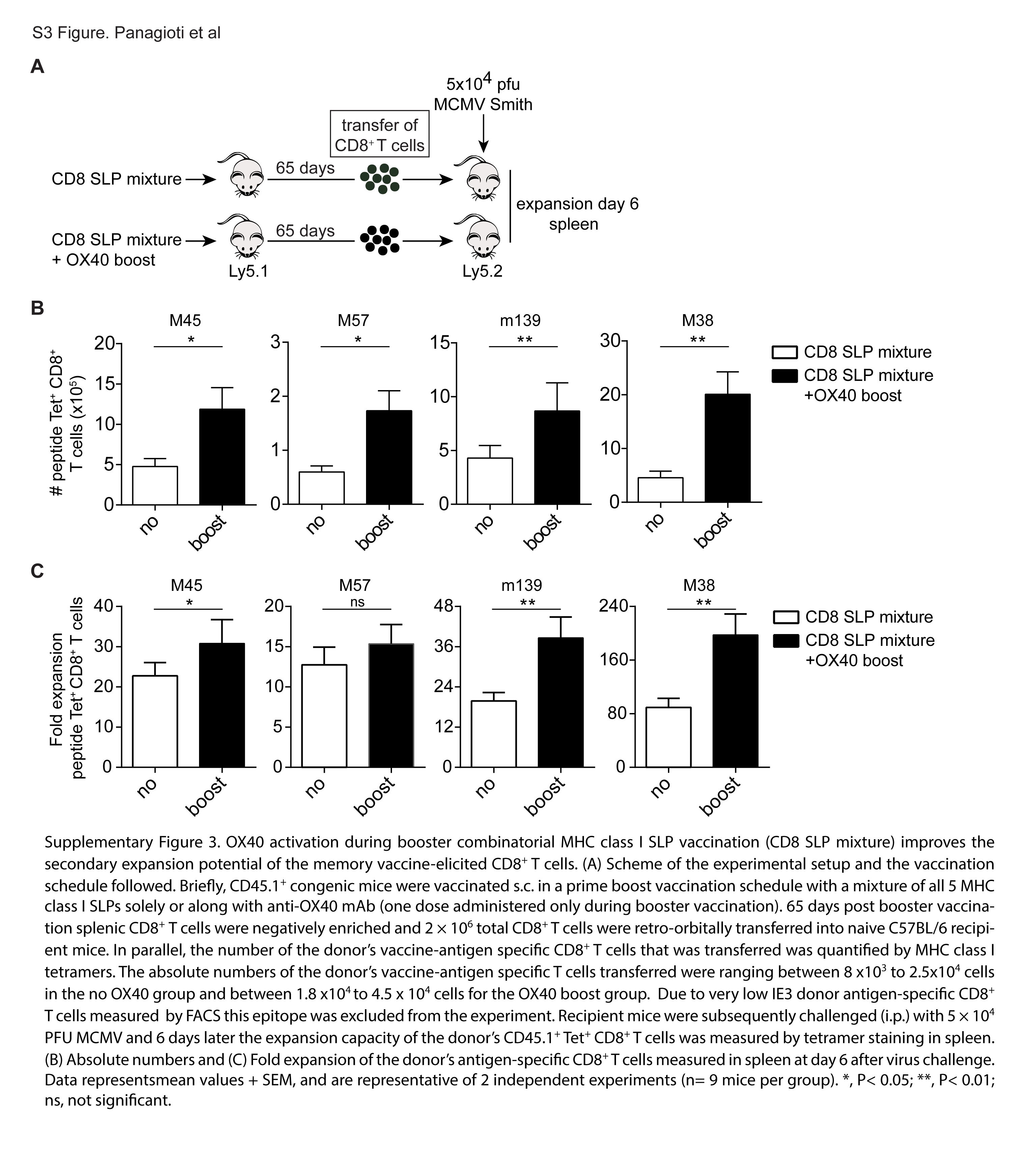

Supplement: Supplementary file 4 [file image_3.tif]

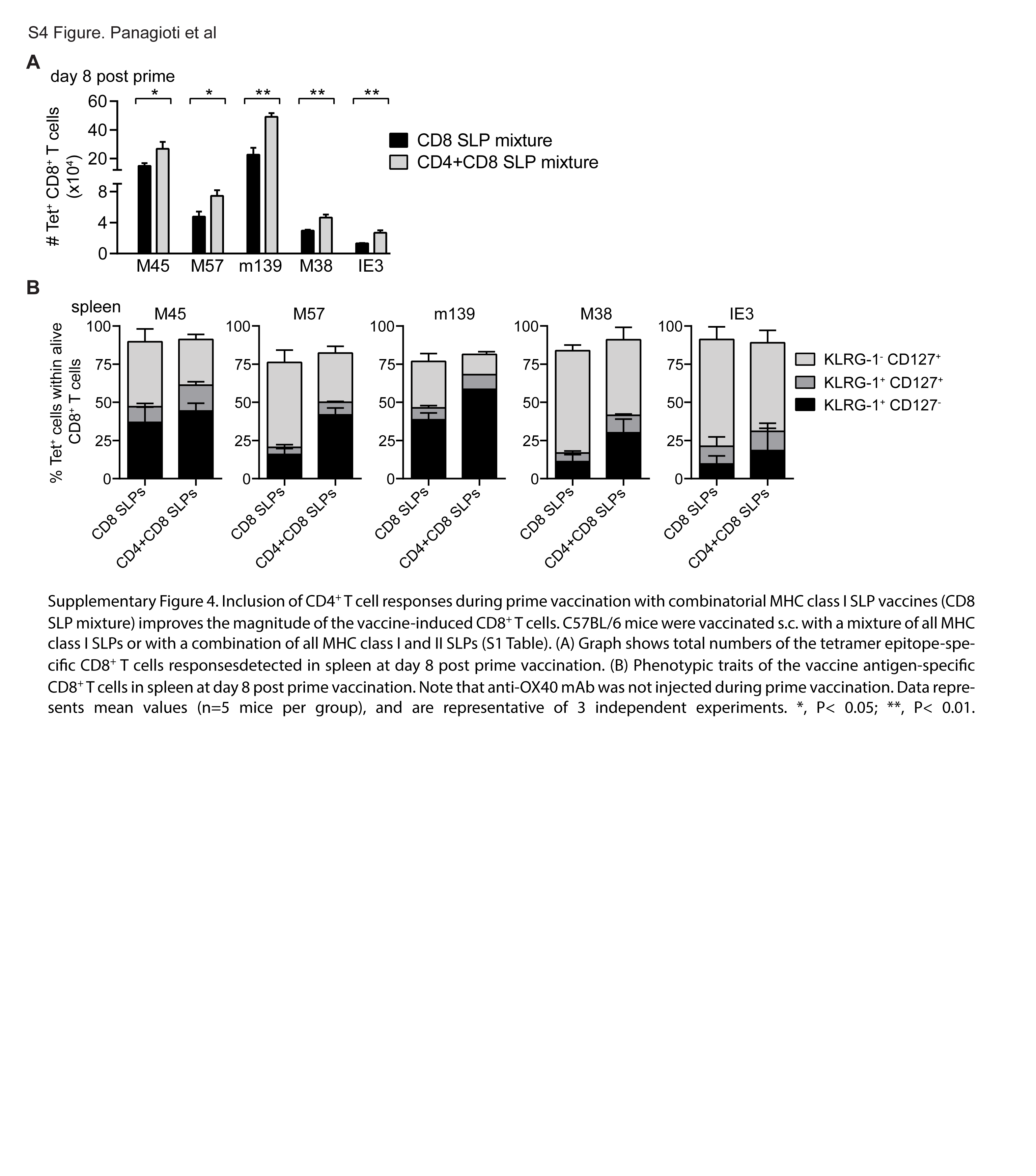

Supplement: Supplementary file 5 [file image_4.tif]

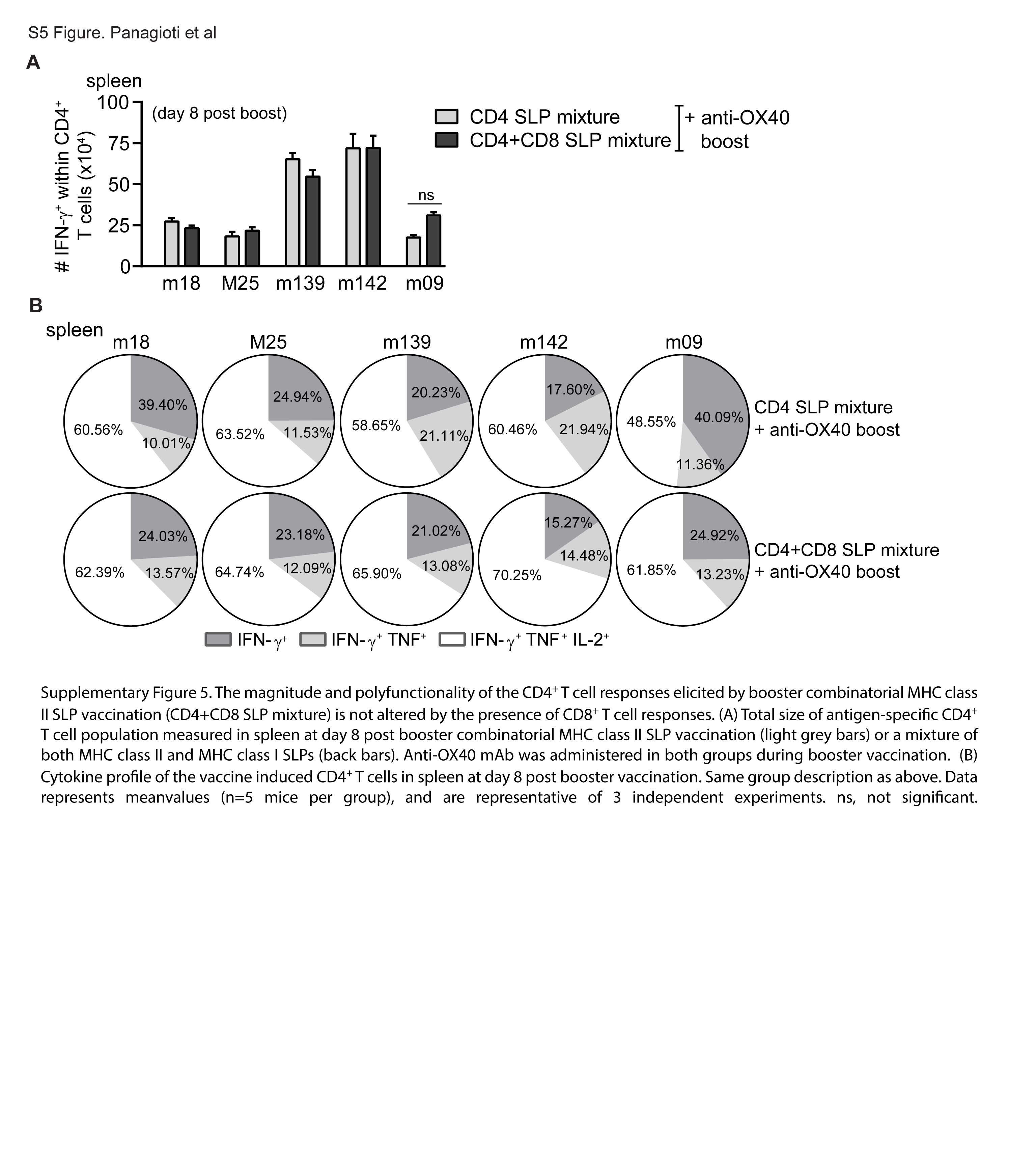

Supplement: Supplementary file 6 [file image_5.tif]

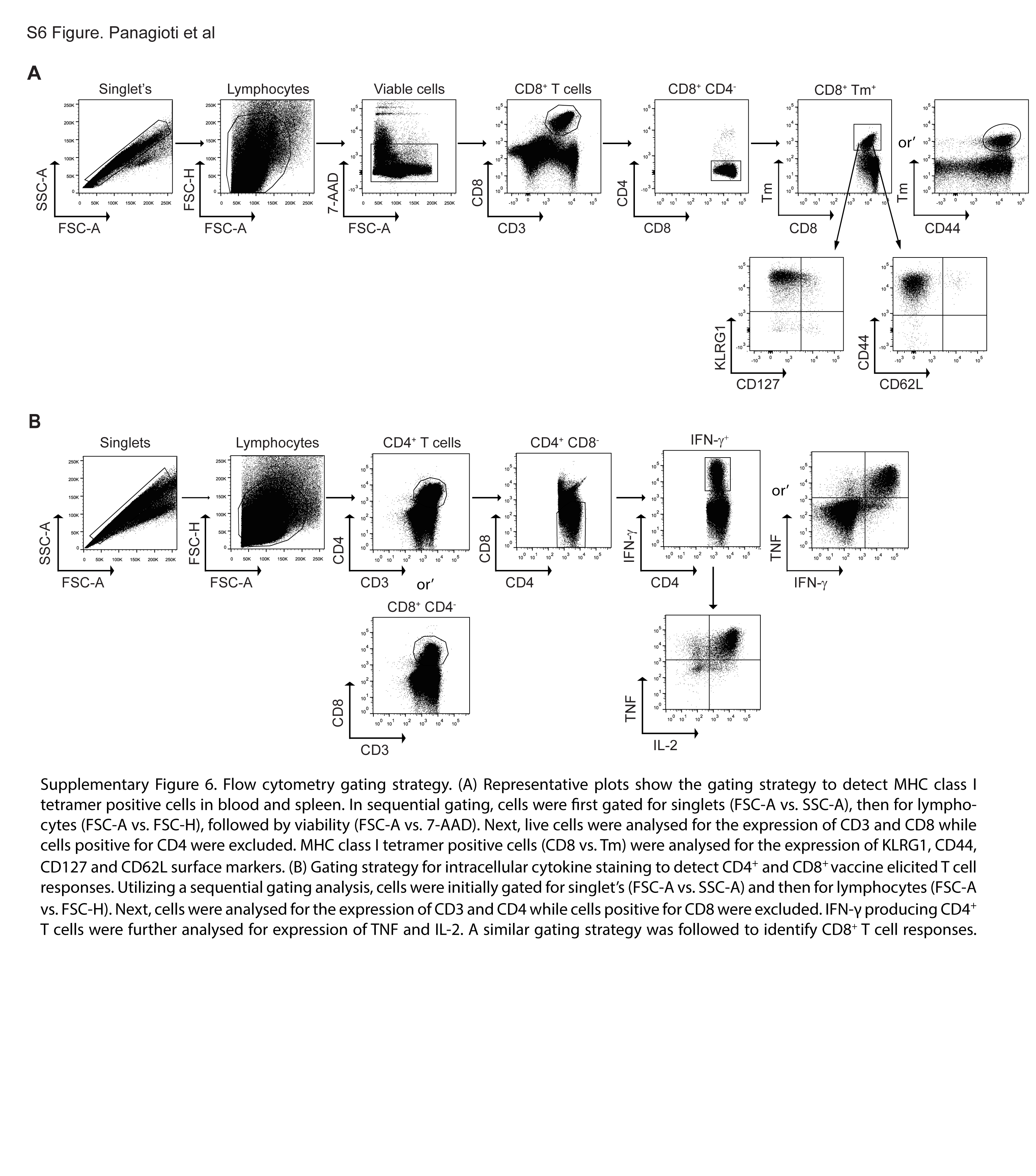

Supplement: Supplementary file 7 [file image_6.tif]
